# The Detection Method of Potato Foliage Diseases in Complex Background Based on Instance Segmentation and Semantic Segmentation

**DOI:** 10.3389/fpls.2022.899754

**Published:** 2022-07-05

**Authors:** Xudong Li, Yuhong Zhou, Jingyan Liu, Linbai Wang, Jun Zhang, Xiaofei Fan

**Affiliations:** ^1^State Key Laboratory of North China Crop Improvement and Regulation, Baoding, China; ^2^College of Mechanical and Electrical Engineering, Hebei Agricultural University, Baoding, China

**Keywords:** potato foliage disease, convolutional neural network, image recognition, instance segmentation, semantic segmentation

## Abstract

Potato early blight and late blight are devastating diseases that affect potato planting and production. Thus, precise diagnosis of the diseases is critical in treatment application and management of potato farm. However, traditional computer vision technology and pattern recognition methods have certain limitations in the detection of crop diseases. In recent years, the development of deep learning technology and convolutional neural networks has provided new solutions for the rapid and accurate detection of crop diseases. In this study, an integrated framework that combines instance segmentation model, classification model, and semantic segmentation model was devised to realize the segmentation and detection of potato foliage diseases in complex backgrounds. In the first stage, Mask R-CNN was adopted to segment potato leaves in complex backgrounds. In the second stage, VGG16, ResNet50, and InceptionV3 classification models were employed to classify potato leaves. In the third stage, UNet, PSPNet, and DeepLabV3+ semantic segmentation models were applied to divide potato leaves. Finally, the three-stage models were combined to segment and detect the potato leaf diseases. According to the experimental results, the average precision (AP) obtained by the Mask R-CNN network in the first stage was 81.87%, and the precision was 97.13%. At the same time, the accuracy of the classification model in the second stage was 95.33%. The mean intersection over union (MIoU) of the semantic segmentation model in the third stage was 89.91%, and the mean pixel accuracy (MPA) was 94.24%. In short, it not only provides a new model framework for the identification and detection of potato foliage diseases in natural environment, but also lays a theoretical basis for potato disease assessment and classification.

## Introduction

Potato is one of the world's four important food crops, one of the 10 most popular nutritious and healthy foods, as well as a high-yield crop with developmental prospects. Due to its high yield and stability, wide adaptability, full nutritional content, and long industrial chain, it has been highly valued in the world (Qu et al., 2005). The early blight and late blight, as the most destructive foliage diseases of potato crops (Tsedaley, 2014; Yellareddygari et al., 2018), could cause major losses in most potato-growing areas in the world. On potato leaves, late blight appears as light green or olive green areas that rapidly turn brownish-black, water-soaked, and oily. Likewise, early blight is round or irregular, which shows dark brown or black spots. Overall, early blight and late blight can occur in all stages of potato growth (Da Silva Silveira Duarte et al., 2019). To control and prevent diseases effectively and timely, it is of great significance to identify and detect the diseases of potato leaves.

In general, the traditional diagnosis of crop diseases is performed by experienced experts, but manual diagnosis is inefficient, subjective, and unsuitable for large regional scenarios. Besides, traditional diagnostic techniques of crop diseases tend to include polymerase chain reaction (PCR), fluorescence *in situ* hybridization (FISH), enzyme-linked immunosorbent assay (ELISA), thermal imaging, and hyperspectral imaging (Fang and Ramasamy, 2015; Xie et al., 2015; Madufor et al., 2018). In the real-life production, farmers need simple, rapid, and accurate ways to identify potato diseases. Therefore, it is crucial to develop a fast, low-cost, time-saving, and labor-saving automatic identification system for potato diseases.

With the advancement in computer vision, artificial intelligence, and machine learning technology, it has promoted the development and implementation of automatic disease recognition technology. For example, Adhikari et al. (2018) used Fast R-CNN (Ren et al., 2017) and R-FCN (Fuentes et al., 2017) to detect diseases of fruit trees, vegetable crops, and other crops, and confirmed good results. In addition, Zhang et al. (2018) used the PlantVillage dataset combined with transfer learning to identify nine tomato diseases. Among them, the models with ResNet as the backbone network have the best recognition effect, with an accuracy of 97.28%. Furthermore, Cheng et al. (2017) used ResNet and AlexNet to identify crop pests, and proved that ResNet101 could achieve the best results, with an accuracy of 98.67%. Khan et al. (2020) proposed a classification method of cucumber foliage disease, which was based on an improved saliency method and deep feature selection. Compared with the existing single-feature selection methods, the deep feature selection method has better performance. To identify cucumber leaf lesions, Wang et al. (2021) put forward a network model fused with UNet and DeepLabV3+, and verified that semantic segmentation has achieved good results for leaf lesions. Apart from that, Fan and Li (2019) proposed a detection method based on key feature points, which could quickly detect the disease in regions of interest by combining with color and texture features. Although this method recognizes 10 types of potato diseases with high speed and high accuracy, it does not have good performance for the recognition in complex environment. Brahimi et al. (2017) trained a convolutional neural network (CNN) composed of nine tomato diseases, with the accuracy of the final model reaching 99.1%. Then, Yang et al. (2020) proposed a potato disease leaf recognition method based on the combination of deep CNN and composite feature dictionary, adopted Faster R-CNN to detect the disease areas, and constructed a composite feature dictionary through extraction of image features. The disease recognition model was trained by support vector machine, and its average recognition accuracy could reach 84.16%. Nevertheless, the image background was relatively simple. To solve the difficult problem of locating and identifying typical potato disease regions under natural conditions, Xiao and Liu (2017) put forward an adaptive feature fusion and rapid recognition method for typical potato diseases. As proved by the recognition experiment of three typical potato diseases, the average recognition rate of the modified adaptive feature fusion method is at least 1.8 percentage points higher than that of the traditional adaptive method. Meanwhile, the average recognition rate of the recognition method is 95.2%, but it is slower than that of deep learning. Additionally, Krishnaswamy Rangarajan and Purushothaman (2020) achieved good results in classifying eggplant diseases, used multiclassification support vector machine (MSVM), and adopted VGG16 as a feature extractor in the eighth convolutional layer. Combining visual object recognition with language generation models, the detailed information about plant anomaly symptoms and scene interactions could be generated (Fuentes et al., 2019). In the task of identifying tomato pests and diseases, the accuracy of the method achieved 92.5%.

Previous studies have applied deep learning technology to the detection, segmentation, or classification of different crop diseases. Beyond that, some studies have proposed to classify different diseases that are found in leaves, and the accuracy rate is generally >90%. At present, there are the following problems in the crop disease recognition and disease spot detection: (1) The image collection in previous studies was often a single leaf, and there were few studies on the segmentation of images containing multiple leaves. (2) Traditional recognition methods have poor recognition rate for plant foliage disease. (3) The effect of plant leaf disease identification on small targets is poor.

Based on the existing research, this study proposed a method of detecting potato diseases in a complex background, which combines instance segmentation, classification model, and semantic segmentation. The main contents of this study are as follows:

(1) A three-stage potato leaf disease detection model based on deep learning was proposed. While segmenting the potato leaves and diseases accurately, this model could provide a basis for establishing a potato leaf disease detection system.
(2) By adopting the three-stage model of instance segmentation, classification model, and semantic segmentation, the advantages of each model were explored. Compared with single model detection, the three-stage model in this study has good performance.
(3) The detection of potato leaf diseases in complex backgrounds was achieved, and the percentage of disease area to leaf area was calculated from the segmented disease area. Overall, this experiment could provide a technical basis for the classification and accurate control of plant diseases in the future.

## Materials and Methods

### Data Collection

**Figure 1:**
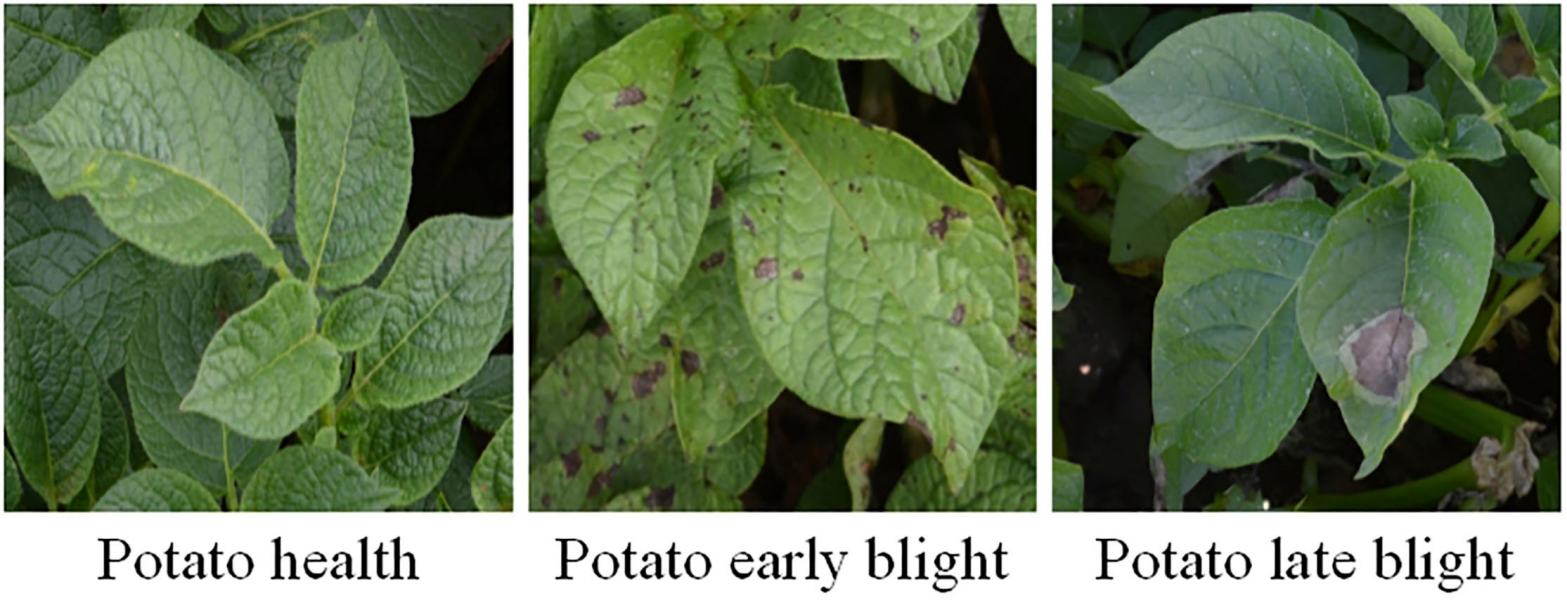
Images of potato leaves.

In this study, potato leaves were collected at the potato experimental site of Hebei Agricultural University, which was a representative planting site in northern China (Weichang and Fengning, Chengde City, Hebei Province). Besides, Nikon D7100 camera with a resolution of 6,000 × 4,000 pixels was used to photograph potato leaves, and it was set to close-up mode with automatic adjustment of focus, aperture, and white. The distance between the camera and the potato plant was about 50 cm, and the images were collected in a vertical manner. The three types of potato leaves are displayed in Figure 1.

### Data Processing

**Figure 2:**
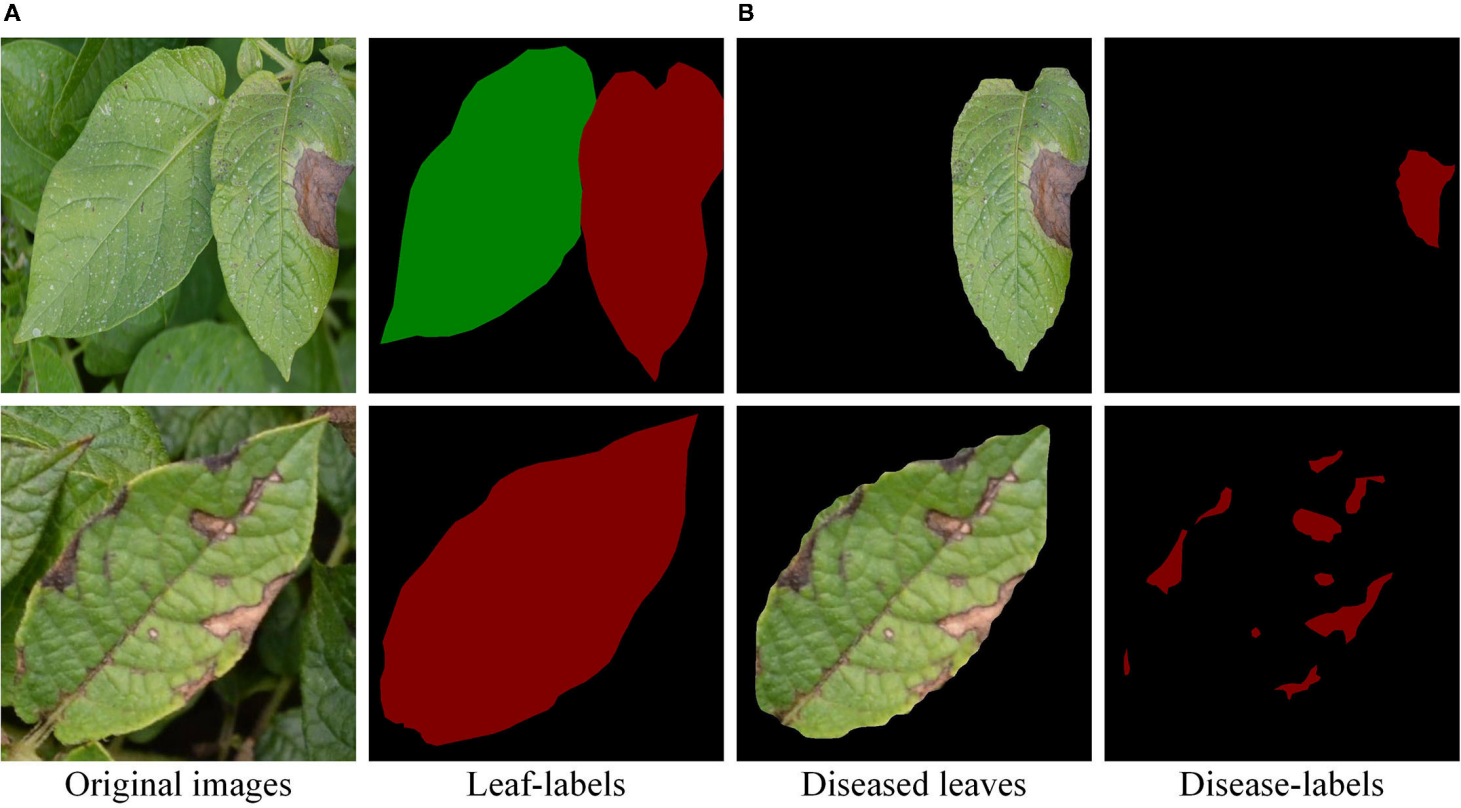
Leaf-labels and disease-labels. **(A)** The individual leaf separated from the complex background. **(B)** The leaf scab was marked.

A total of 500 original images had been collected, including healthy leaves, early blight leaves, and late blight leaves. The size of the original images was adjusted to 800 × 800 pixels. Then, the leaves and diseases were marked by the labelme software. As shown in Figure 2A, the mask images were generated. Apart from that, the accuracy of the model was evaluated by the mask image marked manually. Specifically, the experimental method in this study was divided into three stages. In the first stage, the 400 images were divided into the training set and validation set, respectively, according to the ratio of 4:1 and test set with 100 images after training. The second stage uses image enhancement to obtain 1,800 images, which are divided into training set and validation set according to the ratio of 4:1. The test set consists of 150 original images, including 50 pieces of each of the three types of leaves. In the third stage [as shown in Figure 2B], a total of 632 labeled early blight leaves and late blight leaves images were divided into training set and validation set of the semantic segmentation model in a ratio of 4:1. The test set consists of 50 original images.

### Data Enhancement

Convolutional neural networks require enough data, and the training accuracy of the model could be increased by the amount of data. Therefore, in the second stage of this experiment, the samples were enhanced by image rotation. In addition, the original images were rotated according to the probability of 0.8, with the maximum left-hand angle of 10 and the maximum right-hand angle of 10. In addition, the left and right images were swapped according to the probability of 0.5. The images were zoomed in and out in accordance with the probability of 0.8. In brief, these image enhancement methods simulate the changes in the actual image acquisition angle, direction and distance, increase the diversity of training samples, and improve the robustness and generalization of the model.

### Computer Configuration Parameters

Windows 10 operating system was applied in this study. Specifically, the computer memory is 16 GB, the CPU model is Intel Core (TM) i5-10400f, and the frequency is 2.90 GHz. Meanwhile, the graphics processor model is NVIDIA GeForce GTX 1660s, and the video memory is 6 GB. Software environment used in the experiment is Tensorflow and Keras (Python 3.6).

### Model Evaluation Indicators

To test the performance of the model used in this study (e.g., segmentation, classification model, and semantic segmentation), *Precision* (%), Mean Intersection over Union (*MIoU*, %), *Accuracy* (%), and average pixel accuracy (*MPA*, %) were selected as the indicators. To explain the evaluation index formula conveniently, it was assumed that the data set had a total of *k* + 1 categories. Moreover, $\underline{p_{ij}}$ represents the number of pixels that category *i* is predicted into category *j, P*_*ii*_ represents the number of pixels that are predicted correctly, while *P*_*ij*_ and *P*_*ji*_ represent the number of false-negative and false-positive pixels, respectively.

### Precision and Accuracy

In the formula mentioned below, *TP* denotes true positive, *FP* denotes false positive, and *FN* denotes false negative. *Precision* represents the proportion of the correct prediction that is positive to all predictions that are positive. *Accuracy* represents the proportion of all data that are correctly predicted.


$$\begin{array}{l} Precision\text{=}\frac{TP}{TP+FP} \end{array}$$

$$\begin{array}{l} Accuracy\text{=}\frac{TP+TN}{TP+FN+FP+FN} \end{array}$$

### MIoU and MPA

Pixel-based accuracy (PA, %) calculation is the basic index of semantic segmentation performance evaluation, and *MPA* is the average pixel accuracy. The average intersection ratio is a commonly used measurement index for semantic segmentation and target detection, which is often adopted to evaluate the overlap ratio of the predicted object and the target object. Compared with the pixel accuracy, the average intersection ratio will provide more information, such as the completeness of the predicted target and the coincidence with the actual target.


$$\begin{array}{l} \begin{array}{c} MPA=\frac{1}{k+1}\sum_{i=0}^{k}\frac{P_{ii}}{\sum_{j=0}^{k}P_{ij}} \end{array} \end{array}$$

$$\begin{array}{l} \begin{array}{c} MIoU=\frac{1}{k+1}\sum_{i=0}^{k}\frac{P_{ii}}{\sum_{j=0}^{k}P_{ij}+\sum_{j=0}^{k}P_{ji}-P_{ii}} \end{array} \end{array}$$

### Test Model

#### Mask R-CNN Model

A series of region-based CNN algorithms (He et al., 2017; Ren et al., 2017) are the most representative methods in the target detection. Mask R-CNN, as a relatively novel achievement, can classify, identify, and segment the targets in images. In this study, the backbone network that combines ResNet (He et al., 2016) and FPN (Long et al., 2015) was used to extract features of potato leaves. Among them, the ResNet could sequentially extract low-level features (e.g., edges and corners) and high-level features (e.g., leaves and ground), which could form five layers of feature maps in different sizes and dimensions. If the last layer of features in the ResNet network is used as the output of the network, it is difficult to detect the relatively small leaf features due to its low resolution. Therefore, the FPN network was used to fuse the feature maps from the bottom to the high level, and the features extracted from each layer of the ResNet network were fully used. Apart from that, the feature map extracted from the backbone architecture was input to the regional candidate network. The regional candidate network is a typical binary network, the function of which is to divide the image into two categories, namely, the target leaf and the background. Besides, the plant leaves are boxed out separately in boxes that fit the size of the leaves as closely as possible. At this time, only the approximate region containing the target leaves and the background could be distinguished, and it is impossible to conduct detailed species classification and leaf segmentation of the target leaves. Through the region candidate network, one or more regions containing target blades could be obtained, which are input into ROIAlign to pool into a feature map with a fixed size, and then input into two branches, respectively. One of the branch networks performs target leaf identification by means of a region of interest classifier and a border regressor, both of which include one fully connected layer. One fully connected layer acts as the ROI classifier to classify the ROI into specific plant categories, while the other fully connected layer is used as the border regressor to adjust the center point position and aspect ratio of the ROI, to detect the target leaves more accurately. Another branch network is a segmentation mask generation network consisting of a fully convolutional network, which generates a mask of the same size and shape as the target leaf to segment the target leaf image. Finally, the recognition and results are combined to obtain an image that contains the target leaf class and a segmentation mask that is consistent with the size and shape of the target leaf.

#### Classification Model

The essence of the VGG16 model is an enhanced version of the AlexNet structure, which focuses on the depth of the CNN design. In addition, each convolution layer is followed by a pooling layer. VGG16 has five convolution layers, each with two or three convolution layers. To better extract feature information, this experiment uses three convolutional layers per segment. Beyond that, a maximum pooling layer is connected at the end of each segment to reduce the picture size. The number of convolution kernels in each segment is the same, and the closer they are to the fully connected layer, the more are the convolution kernels. At the same time, the number of convolution kernels in each segment is the same. In general, the closer they are to the fully connected layer, the more are the convolution kernels, and the smaller is the corresponding picture size. As for the VGG network, it uses a smaller convolution kernel, which reduces the number of parameters and saves computing resources. Due to the large number of layers, the convolution kernel is relatively small, so that the entire network has a better feature extraction effect.

The InceptionV3 network is a deep convolutional network developed by Google. Compared with the traditional Inception structure, the V3 version used in this study decomposes the large convolution kernel into small convolution kernels. For example, two 3 × 3 convolution kernels are used to replace the original 5 × 5 convolution kernel, which reduces the number of operations of the model. The BN convolutional layer (Batch Normalization) is added to the classification assistant to improve the accuracy of the model, and the Batch Normalization method is used to make the model perform data normalization preprocessing before each iteration training, which avoids each iteration of the network. All will adapt to different data distributions, which greatly shortens the training time of the model.

The ResNet50 model solves the problem that the actual effect becomes worse due to the increase in network depth and width. It is noteworthy that the deep neural network model sacrifices a large amount of computing resources, while the error rate has also increased. This phenomenon is mainly attributed to the fact that as the number of layers of the neural network increases, the disappearance of the gradient becomes increasingly obvious. The ResNet50 model adds the residual structure (i.e., an identity mapping is added), which converts the original transformation function *H*(*x*) into *F*(*x*) + *x*, makes the network no longer a simple stack structure, and solves the problem of gradient disappearance. This simple stack does not add extra parameters and calculations to the network but improves the effect and efficiency of network training.

#### Semantic Segmentation Model

UNet (Ronneberger et al., 2015) is a semantic segmentation network based on FCN (Long et al., 2015), and its network structure is similar to FCN (fully convolutional networks). The first half of the UNet network is feature extraction, and the second half is upsampling. This structure is generally referred to as an encoder-decoder structure. In addition, the input values of this network are 512 × 512 single-channel or three-channel images. The network, as a whole, can be constructed as a codec architecture or as a systolic path and extended path. On the one hand, each step of the contraction path consists of two 3 × 3 convolutions for feature extraction. On the other hand, each step of the expansion path includes an upsampling process of the feature map, which matches and fuses with the feature map starting from the contracted path. The shallower high-resolution layer in the UNet network is used to solve the pixel localization problem, while the deeper layer is adopted to solve the problem of pixel classification.

The main feature of the PSPNet (Zhao et al., 2016) model is the use of the PSP module. The pyramid pooling module proposed in this model can aggregate the contextual information of different regions, so as to improve the ability to obtain global information. As shown by the results of experiments, such *a priori* representation (referring to the structure of PSP) is effective, and has presented excellent results on multiple data sets. The function of the PSP structure is to divide the acquired feature layers into grids of different sizes, and each grid is pooled on average. It achieves the aggregation of contextual information from different regions, thus improving the capacity to obtain global information.

The main body of the Encoder of DeepLabV3+ (Cheng et al., 2017) is DCNN with hole convolution, which can adopt the commonly used classification networks, such as ResNet, followed by Atrous Spatial Pyramid Pooling (ASPP) module with null convolution (Chen et al., 2014). Compared with the conventional convolution, the hole convolution increases the receptive field without changing the feature map, and retains more spatial detail information. The hole convolution injects “holes” into the standard convolution kernel to increase the convolution kernel. Receptive field, hole convolution uses the hole structure to expand the size of the convolution kernel, which can increase the receptive field without downsampling, while retaining the internal structure of the input data. It is mainly for the introduction of multiscale information. Compared with DeepLabV3, V3+ introduces the Decoder module, which further merges the low-level features with the high-level features to improve the accuracy of the segmentation boundary.

#### Three-Stage Model Structure

**Figure 3:**
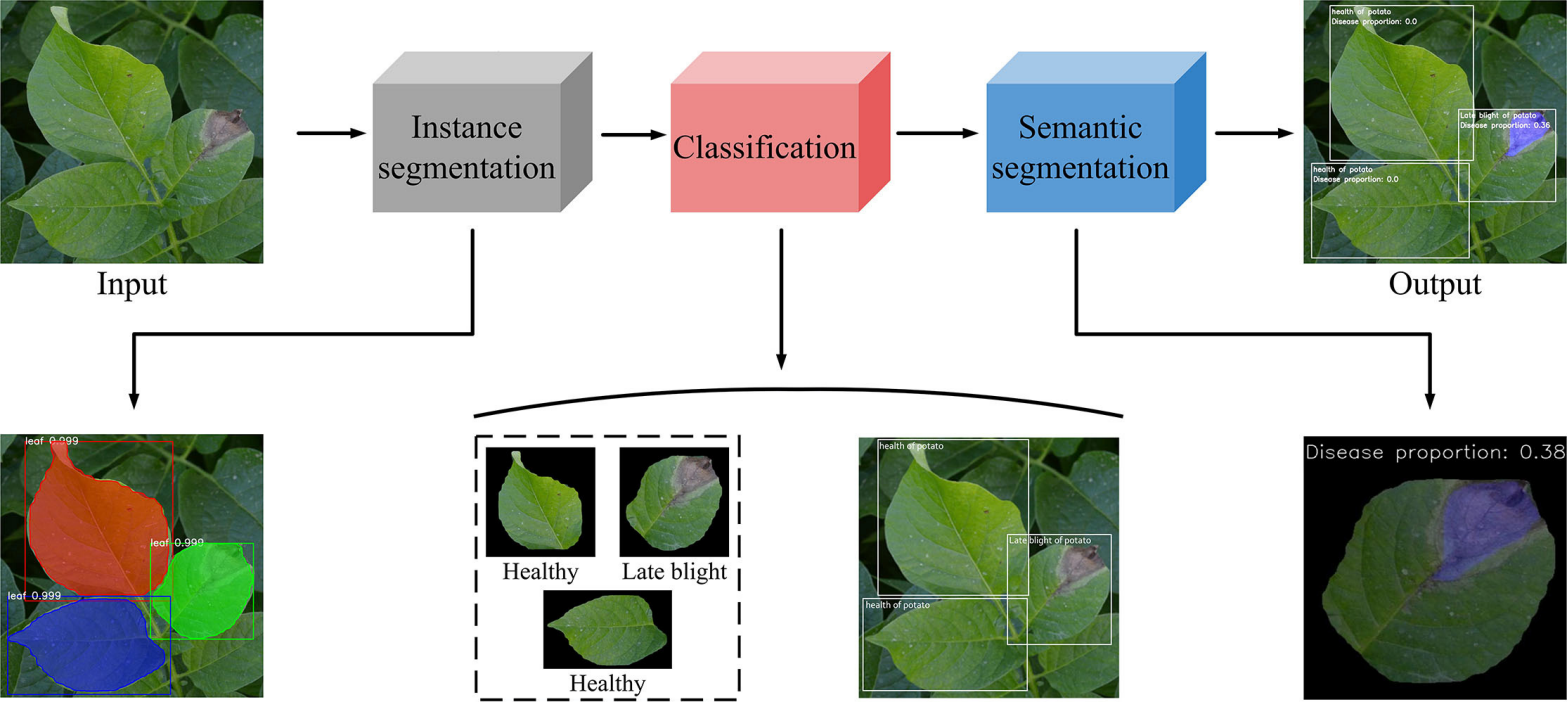
The identification and classification of potato leaf process. This figure shows the whole experimental progress, from the input to the output.

In this study, the potato disease identification consists of four steps (see Figure 3).

(1) In the first stage, potato leaves were segmented by Mask R-CNN from complex background, and the individual leaves were extracted;
(2) The segmented individual leaves were used as the input in the classification model, which could classify healthy, early blight, and late blight leaves;
(3) The single leaf extracted from the second stage was used as the input of the third stage, and the training was carried out through semantic segmentation model;
(4) The disease identified in the semantic segmentation stage was adopted as the index of disease recognition in the classification stage. In addition, the healthy leaves, early blight leaves, and late blight leaves were marked by the instance segmentation model and classification model. The proportion of the disease to the whole leaf was also marked.

## Results

### Mask R-CNN Models

Two different backbone networks, ResNet50 and ResNet101, were used in instance segmentation. Apart from that, 100 pictures were selected to test the models. Table 1 summarizes the results of both networks. It can be observed that the ResNet101 backbone network has a good performance, indicating that a deeper backbone network for features used in Mask R-CNN could obtain the good performance. To better evaluate the accuracy of the whole model, the AP was selected when IoU = 0.5 and IoU = 0.7. Meanwhile, the AP obtained by ResNet50 and ResNet101 was 78.21 and 81.87%, respectively. Furthermore, the Precision obtained by ResNet101 was 97.13%, which was slightly better than that obtained by ResNet50. As ResNet101 has a deeper backbone network, its accuracy in the instance segmentation is higher. For testing 100 images, the two backbone networks need to take 29 and 32 s, respectively. This is because the ResNet101 structure has a deeper network.

**Table 1:** The results of Mask R-CNN model instance segmentation in potato leaves.

**Backbone**	**AP (%)**	**AP_**IoU = 0.5**_ (%)**	**AP_**IoU = 0.7**_ (%)**	**Precision (%)**	**Time/img**
ResNet50	78.21	82.63	84.25	96.73	0.29 s/img
ResNet101	81.87	86.31	85.48	97.13	0.32 s/img

**Figure 4:**
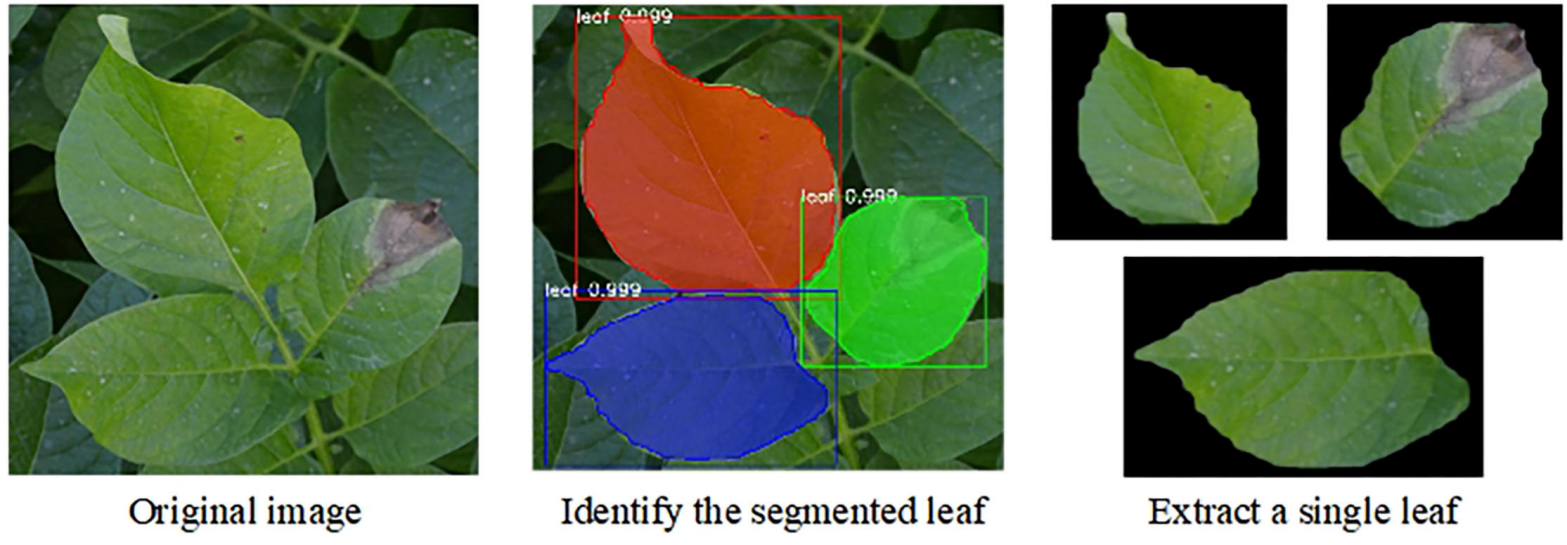
The potato leaves segmented by Mask R-CNN and the single leaf under the black background extracted in the original image.

The results of Mask R-CNN are shown in Figure 4. First, masks of different colors were generated on the leaves. Second, a prediction frame was generated. Finally, the identified leaves were divided into single leaves under the black background, which were used as the input of the second-stage classification model. As displayed in Table 1, the higher precision obtained by the models confirmed that the leaf features could be successfully detected by the models. The two backbone network structures could accurately segment the leaves.

### Classification Models

The single leaf image segmented in the first stage was used as the input in this stage. Beyond that, the leaves were divided into healthy, early blight, and late blight. Additionally, the classification model of this stage utilized the cross-entropy loss function and the Adam optimizer. The batch size was 32, and the learning rate was 0.0001. If the performance of the model did not improve after three epochs, the learning rate would be reduced to continue training, and the iterations would be 150. Table 2 presents the training accuracy of the validation set of the three models.

**Table 2:** Accuracy of the classification model validation in the second stage.

**Model**	**VGG16**	**ResNet50**	**InceptionV3**
Accuracy/%	97.30	95.20	95.70

After the completion of the model training, 50 images were selected as the test set to verify the trained models (see the results in Table 3). Obviously, the Accuracy of the VGG16 network model was up to 95.33%, and the Accuracy ResNet50 and InceptionV3 were slightly lower than those of VGG16.

**Table 3:** Test results of the classification model.

**Model**	**Number of targets (health/early blight/late blight)**	**Number of correct targets (health)**	**Number of correct targets (early blight)**	**Number of correct targets (late blight)**	**Accuracy/%**
VGG16	50/50/50	48	48	47	95.33
ResNet50	50/50/50	48	46	48	94.67
InceptionV3	50/50/50	47	47	46	93.33

### Identification and Detection Models of Early Blight and Late Blight

In the third stage, the single leaf image classified in the second stage was input into the three semantic segmentation models, such as UNet, PSPNet, and DeepLabV3+. Table 4 lists the evaluation indices for the three models, which are obtained after training 150 generations. Obviously, the MIoU and MPA of UNet were higher than those of PSPNet and DeepLabV3+. This is mainly because the early blight is characterized by small area and disease dispersion, which affects the feature extraction of the models. After the completion of model training, 50 pictures of potato leaves with early blight and late blight were selected for testing. Table 4 summarizes the test results of the three network models. It is obvious that the MIoU and MPA of UNet were 89.91 and 94.24%, respectively, which were better than PSPNet and DeepLabV3+. Among them, the MIoU and MPA obtained by DeepLabV3+ were relatively low, which may be due to the addition of hole convolution to the DeepLabV3+ network. Although the receptive field of the convolution layer was increased, some feature information were missed, and the area of some lesions is small, which affects the performance of DeepLabV3+. Compared with PSPNet and DeepLabV3+, UNet uses a more concise network structure and achieves better results. Therefore, UNet provides the feasibility for deployment on resource-constrained mobile devices.

**Table 4:** Comparison of the results in the semantic segmentation models.

**Model**	**MIoU (%)**	**MPA (%)**
UNet	89.91	94.24
PSPNet	86.08	93.19
DeepLabV3+	85.29	88.08

**Figure 5:**
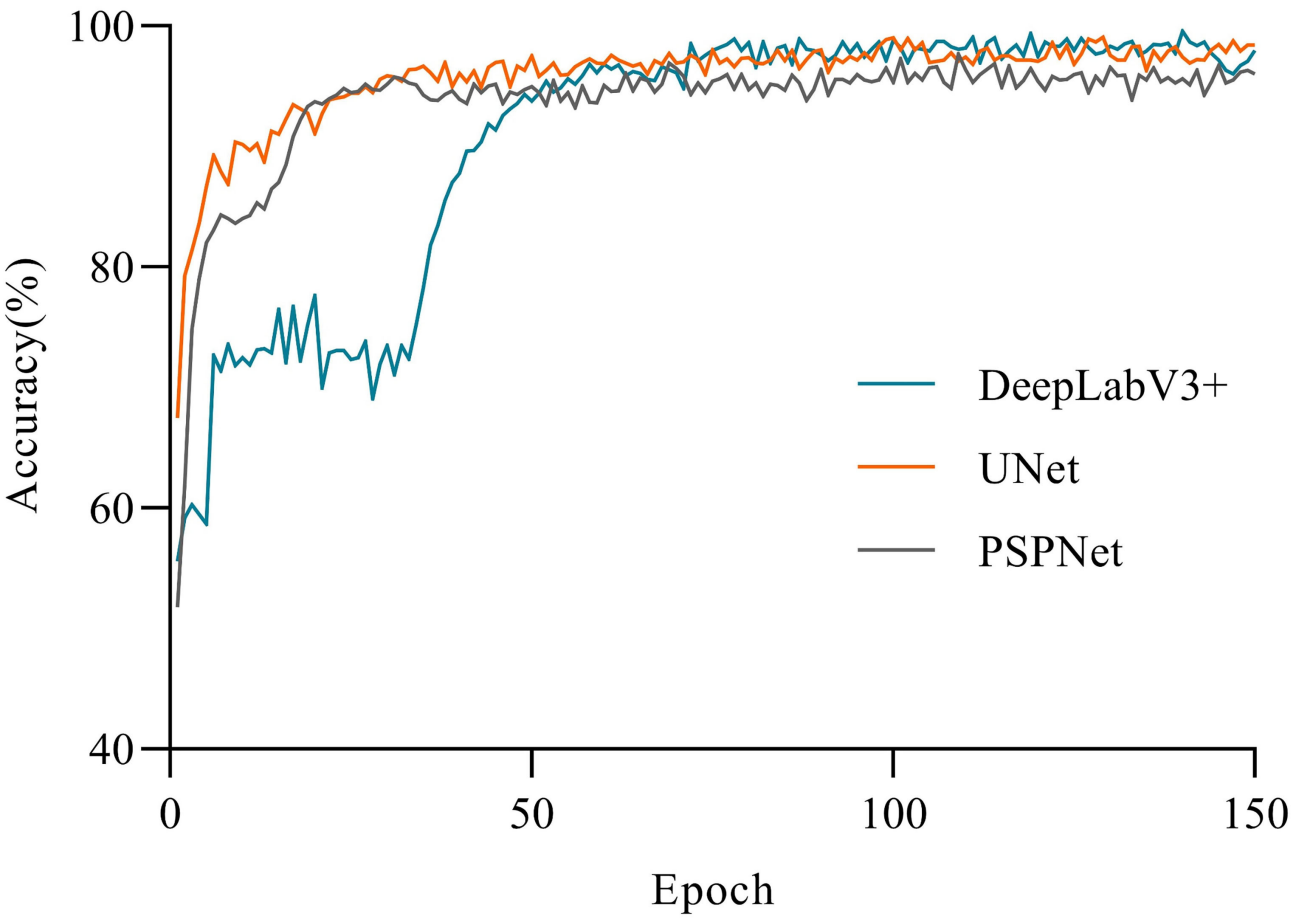
Comparison of the variations of accuracy.

**Figure 6:**
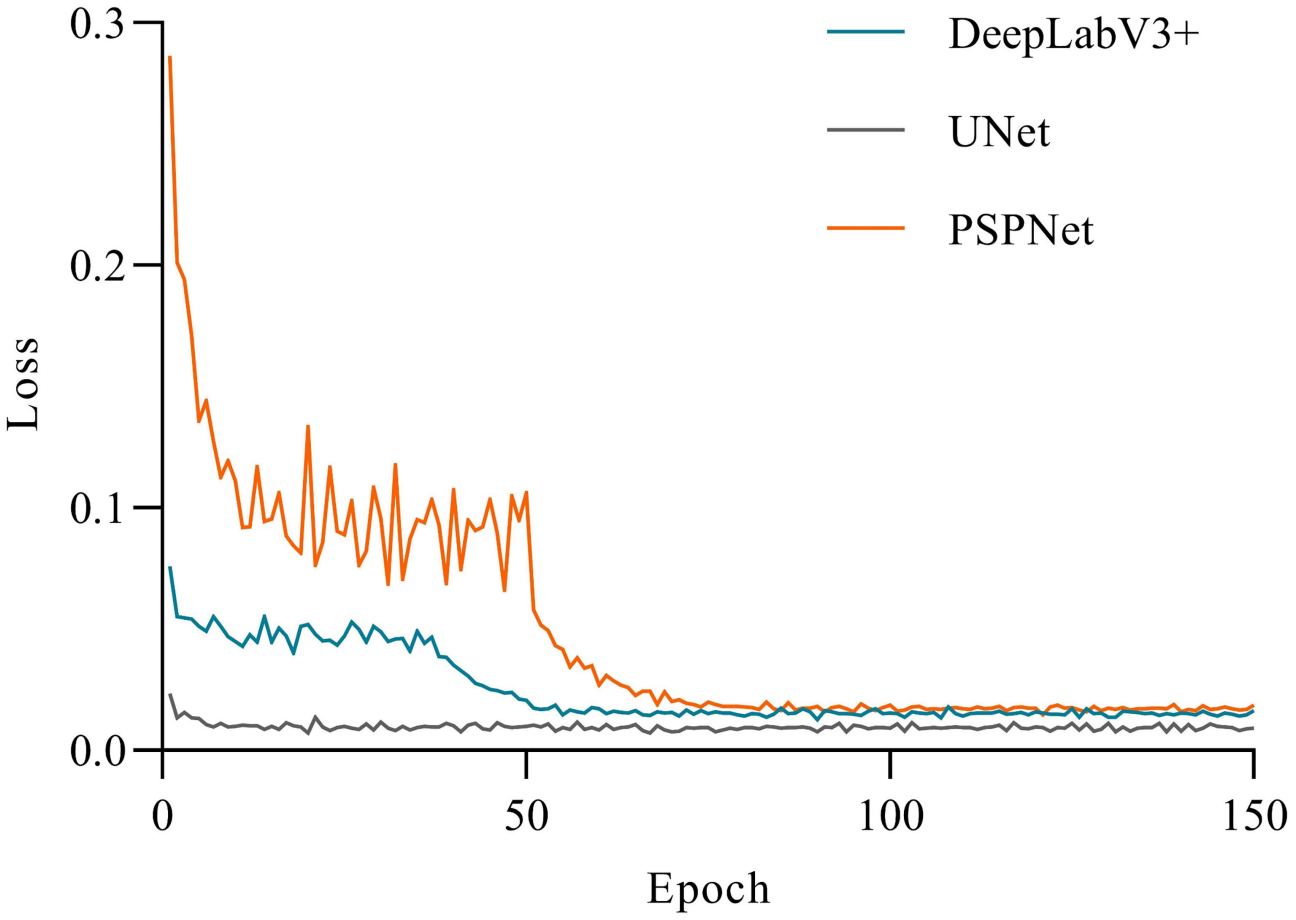
Comparison of the variations of loss.

The accuracy of the three models had a large gap in the initial stage (see Figure 5). UNet achieved higher accuracy at the beginning of the training, and gradually stabilized after 10 epochs. Apart from that, DeepLabV3+ and PSPNet had a low accuracy at the beginning of the training, but DeepLabV3+ reached a relatively high accuracy after 10 epochs, and tended to be stable. Moreover, the first 40 epochs of the PSPNet model were set as the frozen epoch, so that its accuracy began to rise sharply in the 50th epoch. At the same time, PSPNet began to rise after the 40th epoch and gradually stabilized in the 80th epoch, which was closer to UNet at last. As shown in Figure 6, the loss of all models gradually decreased and tended to be stable with the increase of training epochs. Among them, the UNet network model converged faster than other networks and showed lower loss. Besides, the UNet network tended to be stable after 10 epochs. The DeepLabV3+ model gradually stabilized after the 50th epoch, while the PSPNet model had a sharp decline. Apart from that, the loss of PSPNet was stabilized at the 65th epoch, which was very close to DeepLabV3 + after 80 epochs.

**Figure 7:**
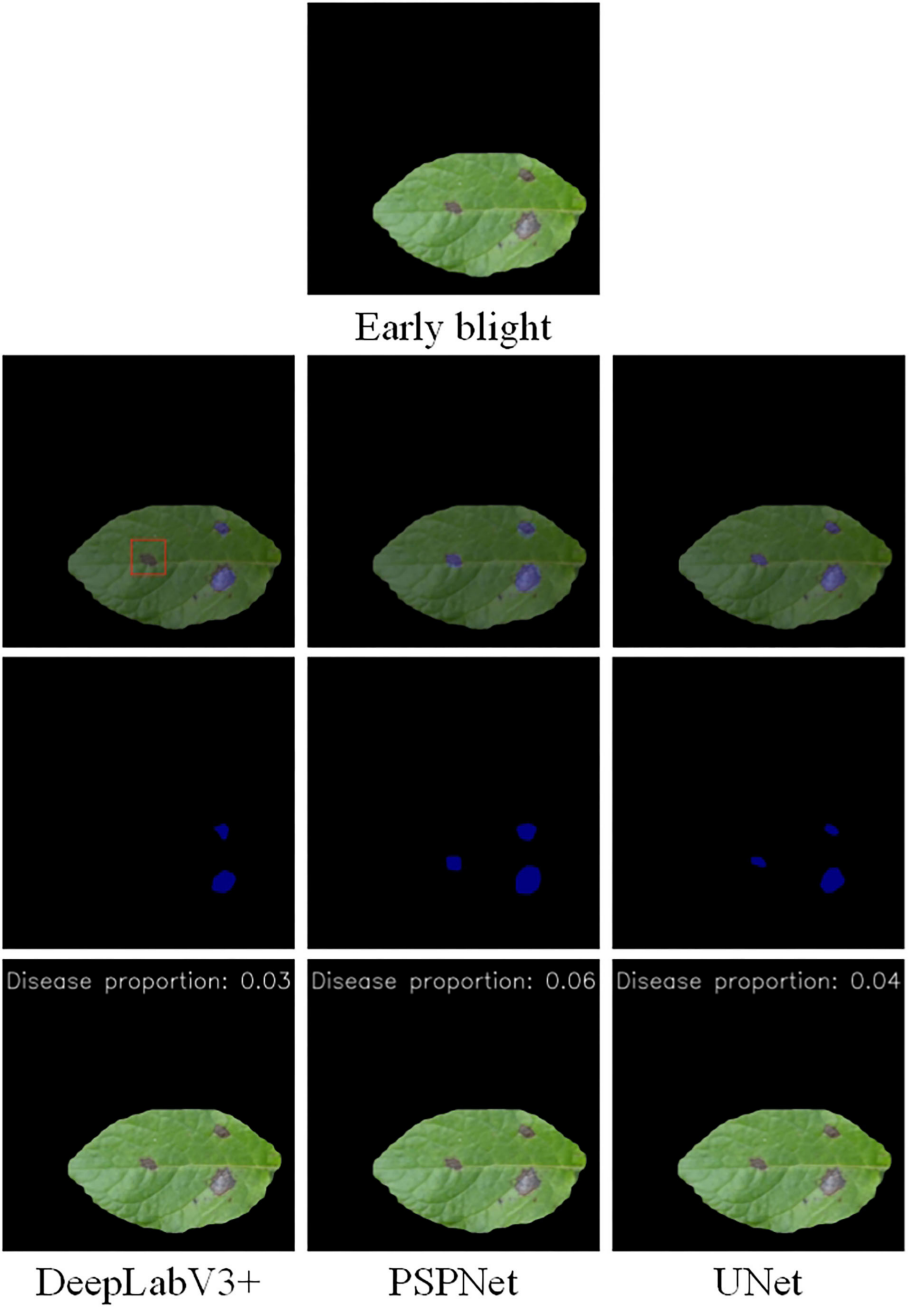
Semantic segmentation results of early blight under the three models.

**Figure 8:**
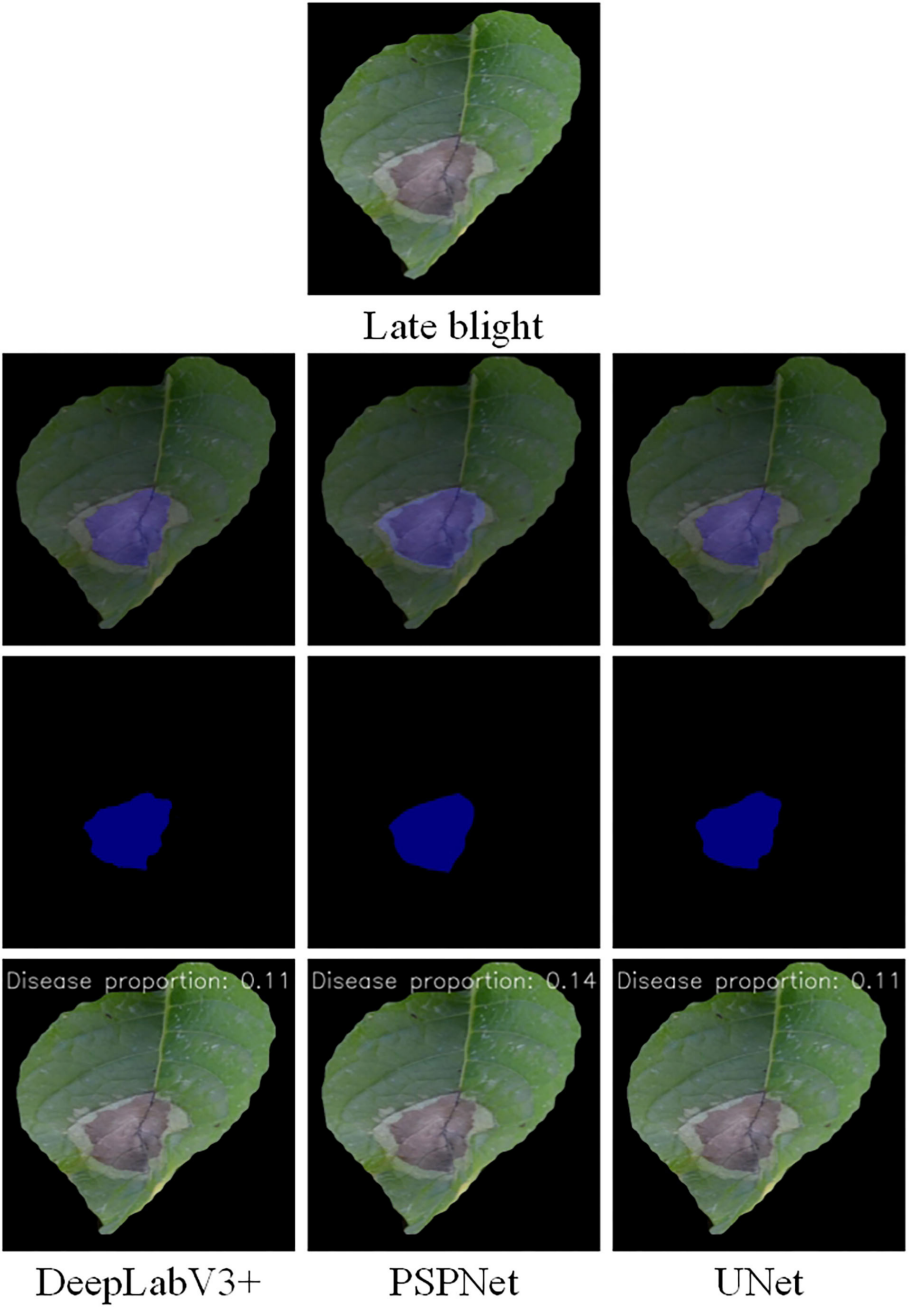
Semantic segmentation results of late blight under the three models.

The disease segmentation results are displayed in Figures 7, 8. In the segmentation of late blight, the three models were relatively accurate and there was not much difference between them. Notably, the proportion of disease areas identified by PSPNet model was the largest. Among them, the edges of the disease area predicted by PSPNet were smoother. These rounded edges can be a factor for the slightly worse performance of PSPNet when compared with the UNet, as some pixels can end up being wrong. The edges predicted by UNet and DeepLabV3+ were more consistent with the actual disease. In the segmentation of the early blight, the disease areas segmented by UNet and PSPNet models were closest to the real situation. Meanwhile, the disease areas predicted by DeepLabV3+ were incomplete. As shown in Figure 8, the disease in the red box was not marked, so that the predicted disease proportion was far from the other two models.

### Model Test Results

**Figure 9:**
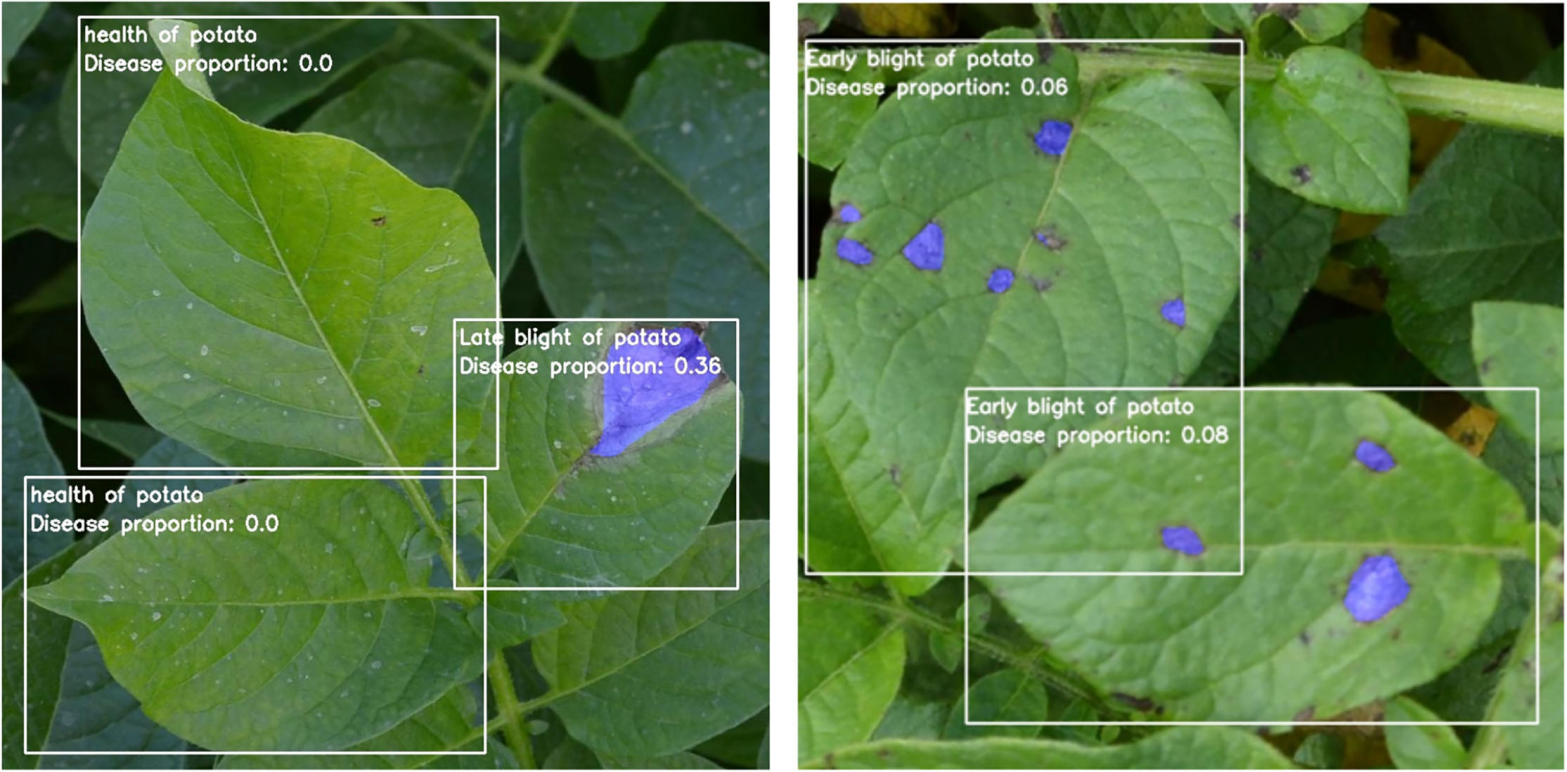
The results of detection and recognition of potato leaves under the three-stage model.

Figure 9 shows the final performance of the three-stage model on potato disease recognition. Initially, an instance segmentation stage processes the input image *via* Mask R-CNN. The instance segmentation stage splits the cropped leaves as the input of the second stage classification model. The classification model classifies leaves into healthy, early blight, and late blight, and takes two diseased leaves as input for the third-stage semantic segmentation. The potato images with complex backgrounds were input into the combined model for detection. In the prediction box, the categories of leaf diseases and the proportion of disease spots were displayed in the upper left corner. In addition, the disease areas were marked on the leaf by calling the model in the semantic segmentation stage.

## Discussion

In summary, the work of this study mainly consists of three parts, namely, leaf segmentation, disease area segmentation, and classification of disease category. Among them, leaf segmentation and disease area segmentation were completed by instance segmentation and semantic segmentation models, respectively. In the first stage, images with complex backgrounds were input into the Mask R-CNN networks, and the leaves without background could be obtained. In the second stage, the leaves without backgrounds were input into the classification networks to distinguish healthy or diseased leaves. In addition, to verify the applicability of the model in real-world scene detection, we further trained the model using the public Plant Village dataset. Finally, the results of this dataset are similar to the data collected in this study, which proves that the classification model used in this study can effectively identify the types of leaves under different disease stages and different degrees of infection. In the third stage, diseased areas based on the labels corresponded to the categories classified in the second stage. In the previous literature, a single model was often used to detect diseases. The experiments in this study have completed the segmentation, classification, and disease spots segmentation of leaves under natural conditions. And this study fuses the three-stage models to realize the detection of the three models on one image. In the final image detection, this study fuses the three models into an input end and an output end, reducing the complex process required for previous detection.

The combination of multi-stage CNN models has been widely applied in various research fields. For instance, Wang et al. (2021) segmented cucumber foliage diseases using a two-stage semantic segmentation model, and the results were better than the single model segmentation. Beyond that, Tassis et al. (2021) identified coffee foliage diseases using a three-stage model, and the AP and MIoU reached 71.90 and 94.25%, respectively. As indicated by the results, compared with the single model, the multi-stage model had a greater improvement in the accuracy of leaf disease detection. Although the three-stage model framework proposed in this study has achieved good results in potato disease detection, there are still some aspects that need to be improved. (1) First, potato early blight disease spots are characterized by small and dense disease area. In this model framework, some disease areas with small area and unclear color differentiation could be identified inaccurately. In the future research, the segmentation accuracy of the little lesions should be improved. (2) Second, in practical potato production, the speed of detection should be increased, and the network structure needs to be improved, so as to shorten the time of model segmentation and better serve the production. (3) In the actual working environment, due to factors, such as large planting area, the efficiency of disease spot detection is high. In this study, the use of mobile phones or cameras to take pictures to collect data will affect the efficiency of actual detection. In the future, we will try to adopt a light-weight CNN structure to reduce the model calculation time, and carry the camera and model program on the drone to achieve rapid detection of the planting area.

## Conclusion

In the first stage, the Mask R-CNN model used two backbone networks, ResNet50 and ResNet101, respectively. The final APs obtained were 78.21 and 81.87%, respectively, and the Precisions were 96.73 and 97.13%, respectively, which achieved accurate segmentation of potato leaves in complex backgrounds.

In the second stage, the classification models were used. Apart from that, the three main networks of VGG16, ResNet50, and InceptionV3 were adopted for experiments. The potato leaves were divided into healthy leaves, early blight leaves, and late blight leaves. Besides, the accuracy of the three networks was 95.33, 94.67, and 93.33%, respectively.

In the third stage, semantic segmentation models PSPNet, UNet, and DeepLabV3+ were used for training of disease region identification. Furthermore, the identification and detection of the early blight and late blight areas were accomplished. The MIoUs were 86.08, 89.91, and 85.29%, respectively, whereas the MPAs were 93.19, 94.24, and 88.08%, respectively, indicating that the segmentation and recognition of potato disease areas were achieved.

In short, this model framework could effectively reduce the impact on potato leaf segmentation in the wild environment, improve the accuracy of disease spot segmentation, and provide technical support for potato leaf disease detection and prevention. The framework presented consisting of three models of CNN can be applied to other crops with some adjustments. In the future, the camera and the program of this study can be mounted on the UAV to realize the application in real scenes.

## Data Availability Statement

The original contributions presented in the study are included in the article/supplementary material, further inquiries can be directed to the corresponding author/s.

## Author Contributions

XL: writing of the original draft. YZ: guiding and supervision. LW: data collection. JZ: proofreading and polishing of the manuscript. JL and XF: editing, supervision, and proofreading. All authors contributed to the article and approved the submitted version.

## Funding

This study was supported by the National Natural Science Foundation of China (32072572), Hebei Talent Support Foundation (E2019100006), the Key Research and Development Program of Hebei Province (20327403D), and the Talent Recruiting Program of Hebei Agricultural University (YJ201847). Detection of crop diseases and insect pests is based on artificial intelligence and multispectral imaging (2021 Shijiazhuang City, the introduction of foreign technology projects).

## Conflict of Interest

The authors declare that the research was conducted in the absence of any commercial or financial relationships that could be construed as a potential conflict of interest.

## Publisher's Note

All claims expressed in this article are solely those of the authors and do not necessarily represent those of their affiliated organizations, or those of the publisher, the editors and the reviewers. Any product that may be evaluated in this article, or claim that may be made by its manufacturer, is not guaranteed or endorsed by the publisher.
